# Fragment-Sized Thiazoles in Fragment-Based Drug Discovery Campaigns: Friend or Foe?

**DOI:** 10.1021/acsmedchemlett.2c00429

**Published:** 2022-11-03

**Authors:** Matic Proj, Martina Hrast, Damijan Knez, Krištof Bozovičar, Katarina Grabrijan, Anže Meden, Stanislav Gobec, Rok Frlan

**Affiliations:** Faculty of Pharmacy, University of Ljubljana, Askerceva 7, Ljubljana 1000, Slovenia

**Keywords:** thiazoles, hit profiling, promiscuous compounds, frequent hitters, privileged scaffolds, fragments

## Abstract

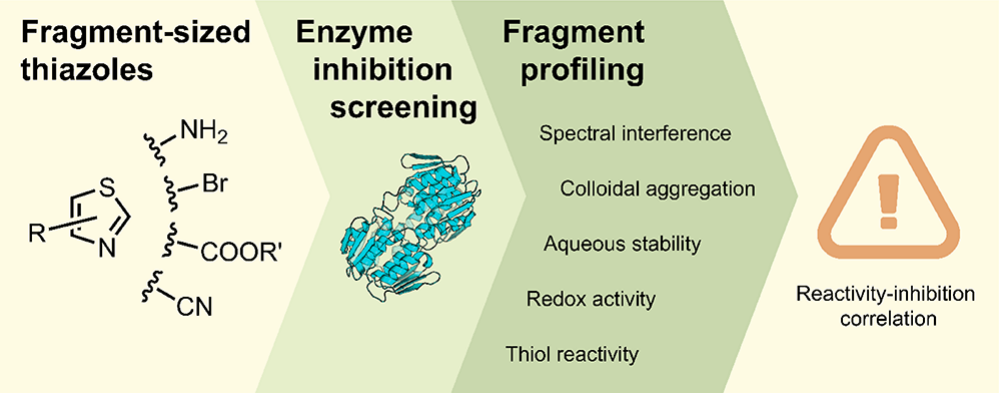

Thiazoles exhibit a wide range of biological activities and therefore represent useful and attractive building blocks. To evaluate their usefulness and pinpoint their liabilities in fragment screening campaigns, we assembled a focused library of 49 fragment-sized thiazoles and thiadiazoles with various substituents, namely amines, bromides, carboxylic acids, and nitriles. The library was profiled in a cascade of biochemical inhibition assays, redox activity, thiol reactivity, and stability assays. Our study indicates that when thiazole derivatives are identified as screening hits, their reactivity should be carefully addressed and correlated with specific on-target engagement. Importantly, nonspecific inhibition should be excluded using experimental approaches and *in silico* predictions. To help with validation of hits identified in fragment screening campaigns, we can apply our high-throughput profiling workflow to focus on the most tractable compounds with a clear mechanism of action.

The thiazole scaffold is present in compounds with various biological activities, such as antiviral, antimicrobial, anticancer, anticonvulsant, antiparkinsonian, and anti-inflammatory activity.^1−3^ It occurs naturally in thiamine (vitamin B1), which is involved in many cellular processes. Thiazoles came up as hits in several of fragment screening campaigns, e.g., Harner et al. described thiazole-based inhibitors of bromodomain of ATAD2,^4^ whereas Tam and colleagues identified phenylthiazole derivatives as new antitubercular lead compounds.^5^ Moreover, the thiazole scaffold was found to be enriched in active fragments from fragment-based screenings at Novartis.^6^ On the other hand, 2-aminothiazoles, for example, are known as frequent hitters.^7^ Therefore, our goal was to determine whether it is worthwhile to pursue thiazole- and thiadiazole-based fragments identified as screening hits for further fragment growing optimization, or rather to divert resources to other fragments.

Fragment-based drug design is an established technique in drug discovery, as demonstrated by six approved drugs that have been derived from fragments.^8^ In an optimal fragment screening campaign, multiple orthogonal biophysical assays are used to confirm target engagement.^9^ Moreover, it is important to identify problematic compounds such as aggregators,^10^ redox cycling compounds,^11^ highly reactive compounds, and other assay interference compounds in the early stages of hit discovery campaigns.^12,13^ Known promiscuous compounds can be filtered out during fragment library design using substructure filters (e.g., PAINS,^14^ Brenk,^15^ Lilly,^16^ and REOS^17^). Alternatively, such compounds can be flagged and retained in the library, yet evaluated thoroughly if identified as hits.^18^ For off-target screens and filters, care should be taken not to remove privileged fragments. These are often desired in fragment-based drug design campaigns, as a single fragment library can be applied to multiple projects and yield hits on unrelated targets. Selectivity can then be tuned in subsequent development steps. In addition, interaction with multiple targets is advantageous when a polypharmacological approach is taken in drug development.^19^

Here, we designed a library of fragment-sized thiazoles and thiadiazoles for screening on enzymes studied in our research group. All compounds were profiled in follow-up experiments adapted for fragment-based screening by biochemical assays (Figure 1). The combination of experimental assays was used to identify thiazoles with nonspecific modes of inhibition and those that interfere with enzymatic inhibition assays.

**Figure 1:**
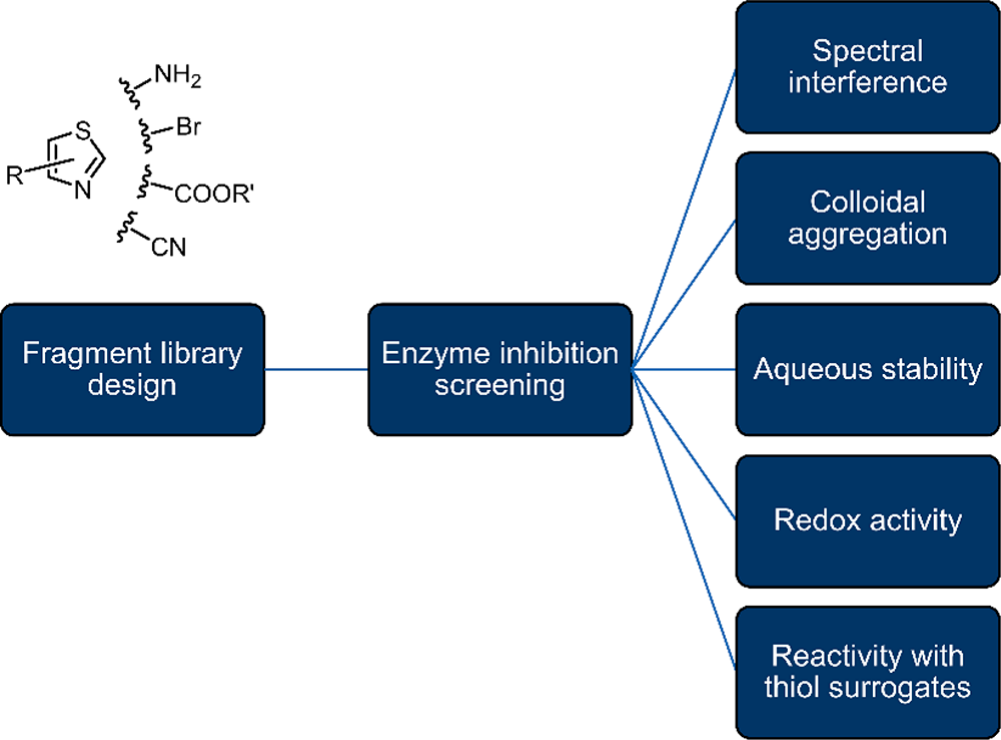
Fragment screening and hit-profiling workflow used in this study.

A substructure search of the ChEMBL database revealed 108 388 bioactive compounds with the thiazole scaffold. Among these, amides are the most abundant, followed by amines, esters, carboxylic acids, bromides, and nitriles (Table 1).

**Table 1:** Number of Bioactive Thiazoles Containing Different Substituents Found in the ChEMBL Database

	substituent position			
substituent	2	4	5	total
any substituent	108 388			108 388
amide	22 179	550	466	23 195
amine (primary)	12 825 (4,062)	627 (479)	500 (396)	13 952 (4,937)
-COOR	31	473	1049	1553
-COOH	12	616	339	967
-Br	61	37	219	317
-CN	23	36	171	230

We designed a focused library of 44 1,3-thiazoles and five 1,3,4-thiadiazoles with different substituents, mainly amines, bromides, carboxylic acids, and nitriles (Figure 2). The substituents were selected based on rapid synthetic and commercial availability while providing access to a variety of chemistries suitable for rapid hit expansion. A set of 25 compounds were sourced from our in-house chemical library, an additional 12 commercially available compounds were purchased, and the sets were enriched by the synthesis of 12 novel compounds. All compounds were evaluated in a cascade of *in vitro* assays.

**Figure 2:**
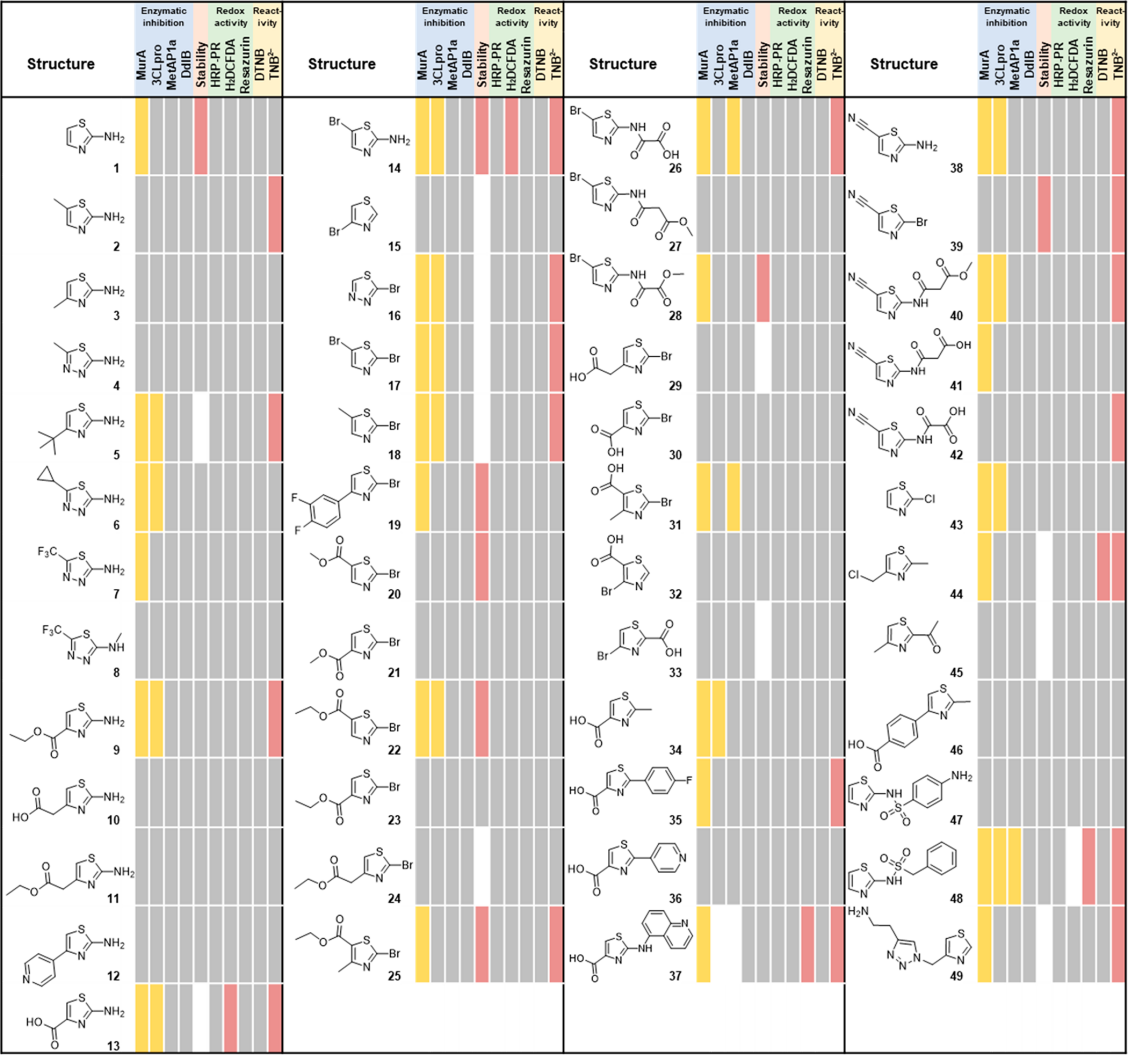
Fragment-sized thiazoles and thiadiazoles that were profiled in four biochemical inhibition assays (MurA, 3CL^pro^, MetAP1a, and DdlB), redox activity (HRP-PR, H_2_DCFDA, Resazurin), thiol reactivity (DTNB and TNB^2–^), and aqueous stability assays. Yellow indicates enzyme inhibition; red indicates redox activity, reactivity, or poor stability; gray indicates lack of inhibition, redox activity, or reactivity. Highlighted in white are compounds with spectral interference for 3CL^pro^, MetAP1a and H_2_DCFDA assays, and compounds with low absorbance that were not evaluated in the aqueous stability assay. A more detailed table is provided in the Supporting Excel file.

First, we determined the inhibitory effect on four unrelated enzymes studied in our research group. Two enzymes contain catalytic Cys residues, namely Cys115 in UDP-*N*-acetylglucosamine enolpyruvyl transferase (MurA) from *E. coli*(20) and Cys145 of 3C-like protease (3CL^pro^) from SARS-CoV-2 virus.^21^ The enzymes containing highly nucleophilic catalytic Cys residues are particularly susceptible to inhibition by electrophilic compounds. Methionine aminopeptidase 1a (MetAP1a) from *Mycobacterium tuberculosis* contains a noncatalytic Cys105 in the active site.^22,23^ In contrast, d-alanine:d-alanine ligase B (DdlB) does not contain surface exposed Cys residues in the active site and close vicinity. To determine the inhibition of MurA and DdlB, we used a colorimetric end point malachite green assay to measure the orthophosphate formed during the enzyme reaction. Inhibition of 3CL^pro^ was determined by kinetic assay using a FRET fluorogenic substrate with a DABCYL–EDANS fluorescence pair. Inhibition of MetAP was monitored by a kinetic assay using a fluorogenic substrate (l-methionine 7-amido-4-methylcoumarin) that is enzymatically hydrolyzed to a fluorescent product 7-amino-4-methylcoumarin. The conditions applied for the screening are routinely used to assay other compounds and chemical libraries for these enzymes. The selected targets and assay technologies provide a range of conditions to evaluate selectivity and highlight potential assay interferences. The threshold for activity in inhibition assays was <50% residual activity (RA) at the concentrations tested (500–625 μM).

Enzymes containing catalytic Cys residues were inhibited by most compounds, i.e., MurA by 26 and 3CL^pro^ by 14 compounds. MetAP1a was inhibited by 3 compounds, and none of the compounds inhibited DdlB, which has no cysteines near the active site (Table 2). Although only four enzymes were examined, a total of nine compounds (**1**, **7**, **19**, **25**, **28**, **35**, **41**, **44**, **49**) were selective for one enzyme, namely MurA (Figure 2). Interference with the malachite green assay system (used in MurA and DdlB inhibition assays) was excluded since none of the thiazoles or thiadiazoles were active in the DdlB inhibition assay. For the fluorimetric 3CL^pro^ and MetAP1a assays, we checked for spectral interference to exclude false positive results. Absorbance values at excitation and emission wavelengths and autofluorescence of the active compounds were measured. Compound **37** showed high absorbance at the excitation wavelengths used for both assays and was therefore classified as a false positive. For some of the most active MurA inhibitors, we determined IC_50_ values and Hill coefficients. The latter could indicate multiple binding for some fragments with Hill coefficient above 1.5 (Supporting Excel file).

**Table 2:** Hit Rate for Each of the Assays Used to Evaluate a Focused Set of 49 Thiazoles and Thiadiazoles^a^

assay	no. of hits (hit rate)
MurA inhibition	26 (53%)
3CL^pro^ inhibition	14 (29%)
MetAP1a inhibition	3 (6%)
DdlB inhibition	0 (0%)
aqueous instability	8 (16%)
HRP-PR redox activity	0 (0%)
H_2_DCFDA redox activity	2 (4%)
resazurin redox activity	2 (4%)
DTNB thiol reactivity	1 (2%)
TNB^2–^ thiol reactivity	19 (39%)

a Compounds with spectral interference were classified as false positives and excluded from the hits.

One of the main causes for false positives in early drug discovery is colloidal aggregation of small molecules.^10^ In MurA, DdlB, and 3CL^pro^ inhibition assays, the detergent Triton X-114 was used to prevent aggregate formation.^24^ In addition, a web application Aggregator Advisor was used to predict the likelihood of compounds as aggregators based on lipophilicity, affinity, and similarity to known aggregators.^25^ None of the compounds are known to aggregate and the similarities to known aggregators were below the threshold. Alternatively, a web application SCAM Detective uses quantitative structure-interference relationship models to detect aggregators.^26^ In this way, two putative aggregators were suggested (**19** and **46**), but only **19** showed inhibition in our assays. In addition, no PAINS alerts were found according to the SwissADME web service.^27^ Therefore, we investigated other mechanisms that could lead to false positive hits in inhibition assays.

The stability of the compounds was determined spectrophotometrically by following the changes in the absorption spectra of the compounds in an assay adapted to the 96-well microplate format. Eight compounds (**1**, **14**, **19**, **20**, **22**, **25**, **28**, **39**) were found to be unstable or intermediately stable in buffer solution (50 mM Tris-HCl pH 7.4, 0.5 mM EDTA) after 60 min (Figure S1). Stability should be considered when evaluating other results, especially for unstable compounds in assays with long incubation times.

Redox activity is a commonly overlooked promiscuous mechanism for false positive results. We used three assays previously optimized for screening large compound libraries.^11^ In the first redox activity assay, H_2_O_2_ generated by redox cycling compounds in the presence or absence of 1,4-dithiothreitol (DTT) was detected with horseradish peroxidase-phenol red (HRP-PR).^28^ Second, compounds generating reactive oxygen species in the presence or absence of the reducing agent tris(2-carboxyethyl)phosphine (TCEP) were detected with a fluorescent probe, 2′,7′-dichlorodihydrofluorescein diacetate (H_2_DCFDA).^29^ Third, free radicals formed by redox cycling of compounds with DTT were detected with resazurin.^30^ Spectral interferences were also determined in the fluorimetric H_2_DCFDA and resazurin assays. Compound **48** showed strong autofluorescence under the conditions used in the H_2_DCFDA assay but did not interfere with the readout of the resazurin assay. As the example of **48** shows, it is beneficial to use multiple orthogonal assays to avoid spectral interference in the determination of redox activity.^31^ Overall, four compounds (**13**, **14**, **37**, and **48**) were redox active in at least one of the assays. The observed redox activity could be related to enzyme inhibition, as the redox active compounds inhibited two or three Cys-containing enzymes.

Nonspecific covalent modification of protein amino acid residues is another plausible and commonly encountered form of promiscuous inhibition, which has to be clearly separated from screening of curated covalent fragment libraries.^32^ We have performed reactivity assays in nonproteinaceous environment to determine whether compounds that inhibit enzymes with catalytic Cys react with thiol surrogates. A thiol-containing colorimetric probe was used as a Cys surrogate in an assay with reduced 5,5'-dithio-bis(2-nitrobenzoic acid) (DTNB).^33^ In this experiment, TNB^2–^ is generated *in situ* from DTNB by the reducing agent TCEP. The consumption of 5-mercapto-2-nitrobenzoic acid (TNB^2–^) is then followed spectrophotometrically at 412 nm to determine the alkylation rate (Figure S2). As we have previously described,^11^ TCEP can be eliminated from the assay in a parallel experiment, since some compounds are known to react with it. To avoid this reaction, TNB^2–^ was used directly in place of DTNB. After completion of the reaction between TNB^2–^ and the compound, TCEP was added to determine the reversibility of the reaction (Figure S3). Elimination of TCEP was crucial in this assay because only compound **44** was reactive in the presence of TCEP and 19 compounds were reactive in the modified assay without TCEP. Moreover, for all reactive compounds except **39** and **44**, the absorbance was restored upon addition of TCEP and the reaction with TNB^2–^ was reversible. The activity and reactivity profiles (Figure 2) revealed that 10 compounds inhibited both MurA and 3CL^pro^ and were reactive in one of the thiol reactivity assays. Another four reactive compounds inhibited MurA. Two of three MetAP1a inhibitors were reactive in one of the thiol reactivity assays. In addition, most of the reactive compounds inhibited at least one of the enzymes (Table 3), from which we can conclude that for our set of compounds inhibition is related to reactivity. On the other hand, reactivity can be problematic for fragment-sized compounds devoid of distinct electrophilic warheads because covalent binding outweighs contributions to binding affinity from other noncovalent interactions.

**Table 3:** Contingency Table Describing the Correlation between Inhibitory Activity in Any of the Enzymatic Inhibition Assays and Thiol Reactivity for Our Library of Thiazoles and Thiadiazoles^a^

	thiol reactivity	
	reactive	not reactive
inhibitory activity	16	10
no inhibitory activity	3	20

a Most of the reactive compounds showed inhibitory activity.

To confirm the reactivity hypothesis for some compounds, we performed inhibition assays for MurA and 3CL^pro^ in the presence of the reducing agent DTT. DTT not only stabilizes the enzyme but also can act as a radical scavenger and react with electrophilic compounds. Indeed, the inhibitory activity in the presence of DTT was abolished for all compounds except **37** for 3CL^pro^. As mentioned earlier, the inhibitory effect of **37** on 3CL^pro^ is false positive because of spectral interference with the fluorescence measurement. When we performed the MurA inhibition assay without the 30 min preincubation, 9 of 26 compounds lost activity, indicating time-dependent inhibition (Figure 2). However, four compounds that inhibited MurA only after 30 min preincubation were found to be unstable or intermediately stable in buffer (**1**, **19**, **22**, and **28**). Considering their low stability, the degradation products formed during the incubation period could be responsible for the inhibition. Seven of the time-dependent inhibitors were selective for MurA, which contains catalytic Cys that is particularly susceptible to electrophilic compounds.

Molecular descriptors derived from quantum-mechanical (QM) calculation have long been used to predict and explain the reactivity of various compounds.^34−37^ Therefore, a number of molecular descriptors have been calculated at the semiempirical (PM7) level (HOMO and LUMO energies, Mulliken electronegativity, molecular electronegativity, Parr Pople hardness, molecular hardness, total nucleophilic superdelocalizability, total electrophilic superdelocalizability, total atom self-polarizability) and DFT (M06-2X-D3/LACVP**++) level (HOMO and LUMO energies, minimal and maximal electrostatic potential (ESP), polarizability, minimal and maximal average local ionization energy (ALIE), Fukui indices, chemical hardness, electrophilicity index, and electronic chemical potential) and correlated with the observed reactivity (Supporting Excel file). No meaningful relationships were observed between any of these descriptors and the reactivity parameters, i.e., the number of flags and, in particular, thiol reactivity (Figure S4). Although the compounds share the thia(dia)zole ring system, they are otherwise quite heterogeneous and no logical mechanism could be demonstrated to explain the reactivity or warrant further QM studies.

In conclusion, we present an example of a high-throughput workflow for profiling screening hits. We designed a small and focused library of thiazoles and thiadiazoles that was evaluated in an assay cascade. In addition to enzymatic inhibition assays, we evaluated redox activity, reactivity, spectral interference, and stability of the fragments. Half of the fragments were flagged in more than one assay, showing a high correlation between biological activity and reactivity. The covalent mechanism of inhibition is suggested for some compounds, as they were inactive in the biochemical assay upon addition of DTT, exhibited time-dependent inhibition, and reacted in the thiol reactivity assay. Molecular descriptors indicative of electrophilicity were calculated at the semiempirical and DFT levels of theory but did not correlate with the observed reactivity profile. Furthermore, no probable consensus mechanism of action emerged, which remains to be elucidated. However, the thiazoles and thiadiazoles studied here have different activity and reactivity profiles, and not all are problematic. Moreover, it can be advantageous if certain fragments are frequent hitters because hits can be obtained even when screening a small fragment library, and the selectivity can be improved in the next steps of fragment growing. Importantly, we do not want to establish a general knockout criterion to exclude thiazole or thiadiazole screening hits from further development, but it is essential to evaluate their reactivity if they prove to be hits. This is particularly important when dealing with proteins that are more susceptible to electrophilic compounds, such as enzymes with a catalytic Cys residue. Nonspecific inhibition of thiazoles and thiadiazoles should be excluded using experimental approaches and *in silico* predictions. As shown in this study, thorough hit profiling is an important step in fragment screening campaigns to highlight potential liabilities of hit fragments.

## Supporting Information Available

Supporting figures; experimental procedures; synthetic procedures and characterization of compounds (PDF)
List of compounds with experimental results and results from quantum-chemical calculations (XLSX)

## Abbreviations

3CL^pro^: 3C-like protease
DdlB: d-alanine:d-alanine ligase B
DTNB: Ellman’s reagent (5,5'-dithio-bis(2-nitrobenzoic acid))
DTT: 1,4-dithiothreitol
H_2_DCFDA: 2′,7′-dichlorodihydrofluorescein diacetate
HRP-PR: horseradish peroxidase-phenol red
MetAP1a: mycobacterial methionine aminopeptidase 1a
MurA: UDP-*N*-acetylglucosamine enolpyruvyl transferase
QM: quantum-mechanical
RA: residual activity
TCEP: tris(2-carboxyethyl)phosphine
TNB^2–^: 5-mercapto-2-nitrobenzoic acid
