# Supplementary material for: Fragment-Sized Thiazoles in Fragment-Based Drug Discovery Campaigns: Friend or Foe?
Source: ACS Med Chem Lett. 2022 Nov 3;13(12):1905–10. doi: 10.1021/acsmedchemlett.2c00429 (PMC9743419; doi:10.1021/acsmedchemlett.2c00429)
Supplement: Supplementary file 1 — ml2c00429_si_001.pdf [file ml2c00429_si_001.pdf]

Supporting information

Fragment-sized thiazoles in fragment-based drug discovery campaigns: friend or foe?

Matic Proj [a], Martina Hrast [a], Damijan Knez [a], Krištof Bozovičar [a], Katarina Grabrijan [a], Anže Meden [a], Stanislav Gobec [a], Rok Frlan [a*]

[a] *University of Ljubljana, Faculty of Pharmacy, Askerceva 7, 1000 Ljubljana, Slovenia*

* *Corresponding author. Rok Frlan, e-mail address: rok.frlan@ffa.uni-lj.si*

Table of contents

1 Supporting figures S2

2 Experimental procedures S6

2.1 ChEMBL substructure search S6

2.2 Enzymatic inhibition assays S6

2.3 Aqueous stability assay S9

2.4 Redox activity assays S10

2.5 Thiol reactivity assays S11

2.6 Quantum-mechanical calculations S12

3 Chemistry S12

3.1 General chemistry S12

3.2 General synthetic procedures S13

3.3 Synthesis and characterization of compounds S14

4 References S19

1 Supporting figures

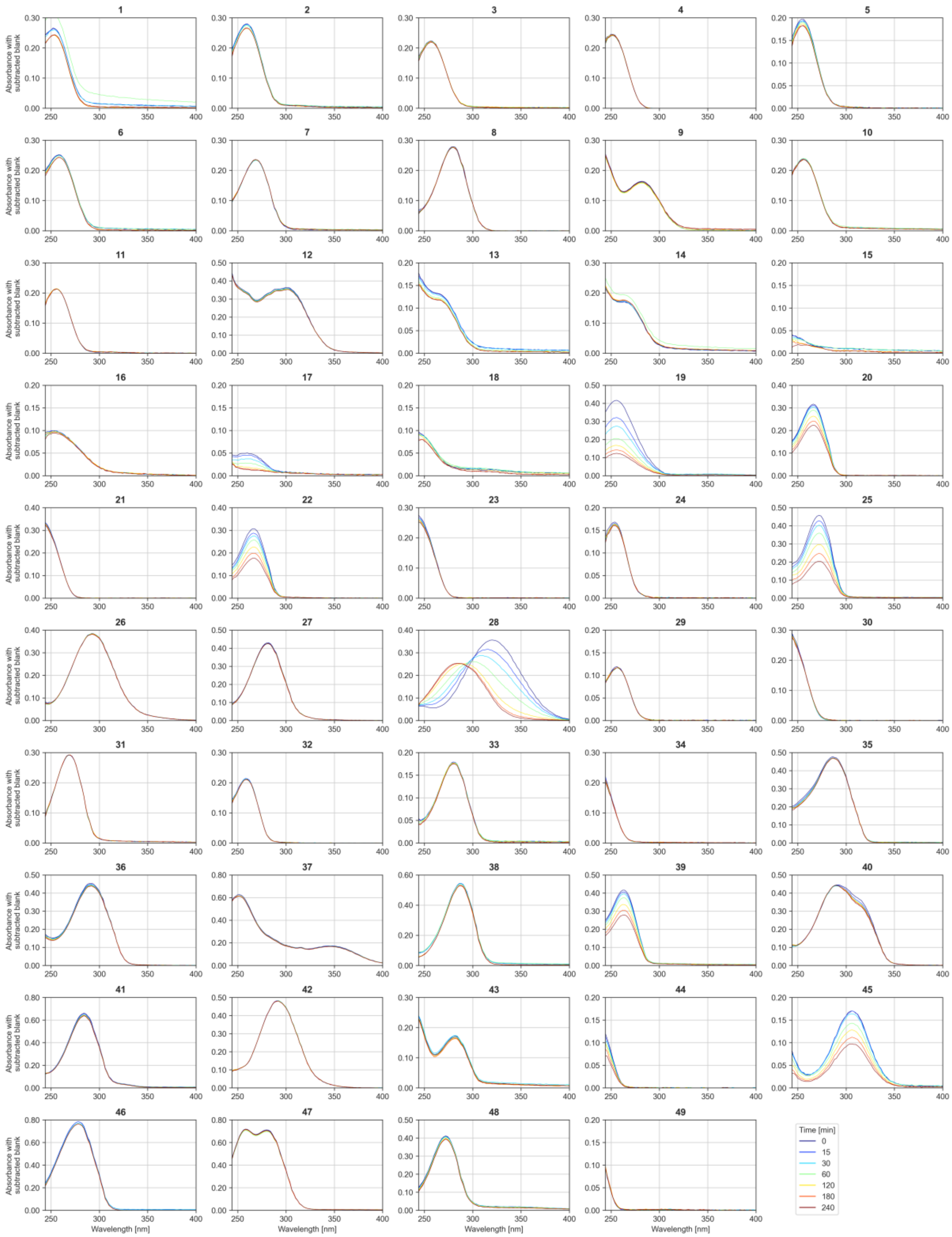

Figure S1. Absorbance spectra for determination of stability in aqueous solution. Significant changes indicate instability (e.g., **28**).

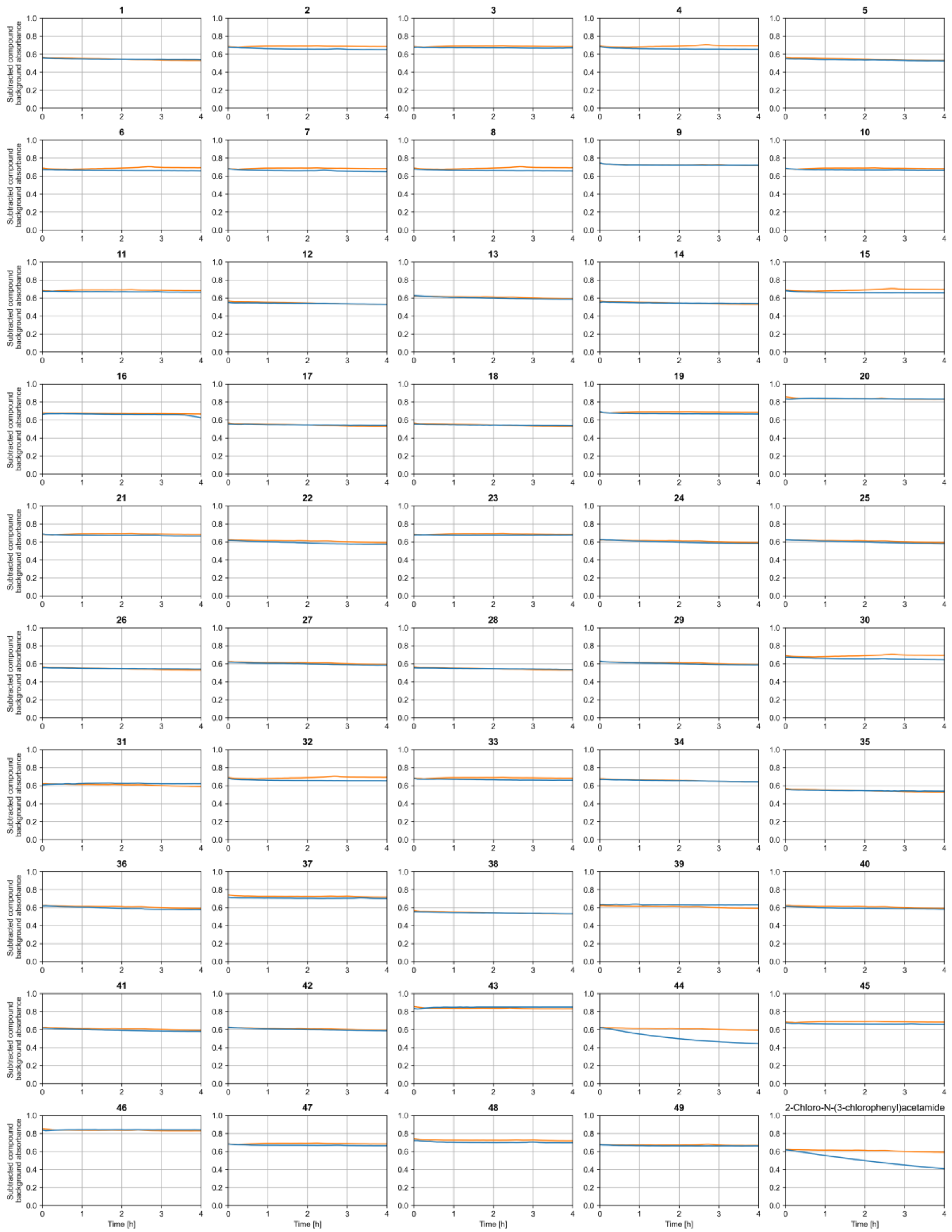

Figure S2. Plots from the DTNB reactivity assay. Compound background absorbance was subtracted from each measurement. Orange lines denote baseline and blue lines denote compounds. 2-Chloro-*N*-(3-chlorophenyl)acetamide was used as a control compound.

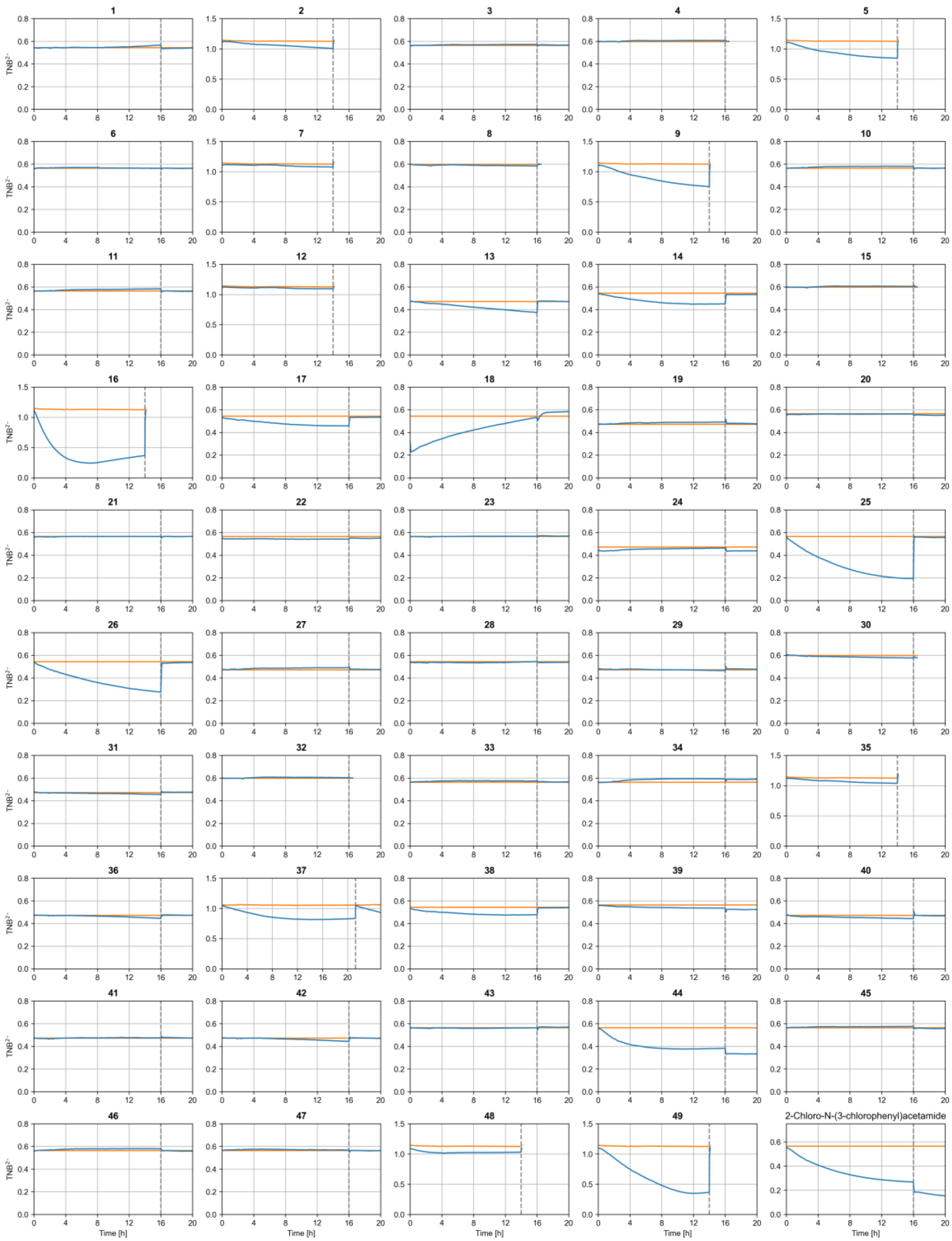

Figure S3. Plots from the TNB^{2-} reactivity assay. Compound background absorbance and baseline drift were subtracted from each measurement. Vertical dashed line denotes time of TCEP addition to determine the reversibility of the reaction. Orange lines denote baseline and blue lines denote compounds. 2-Chloro-*N*-(3-chlorophenyl)acetamide was used as a control compound.

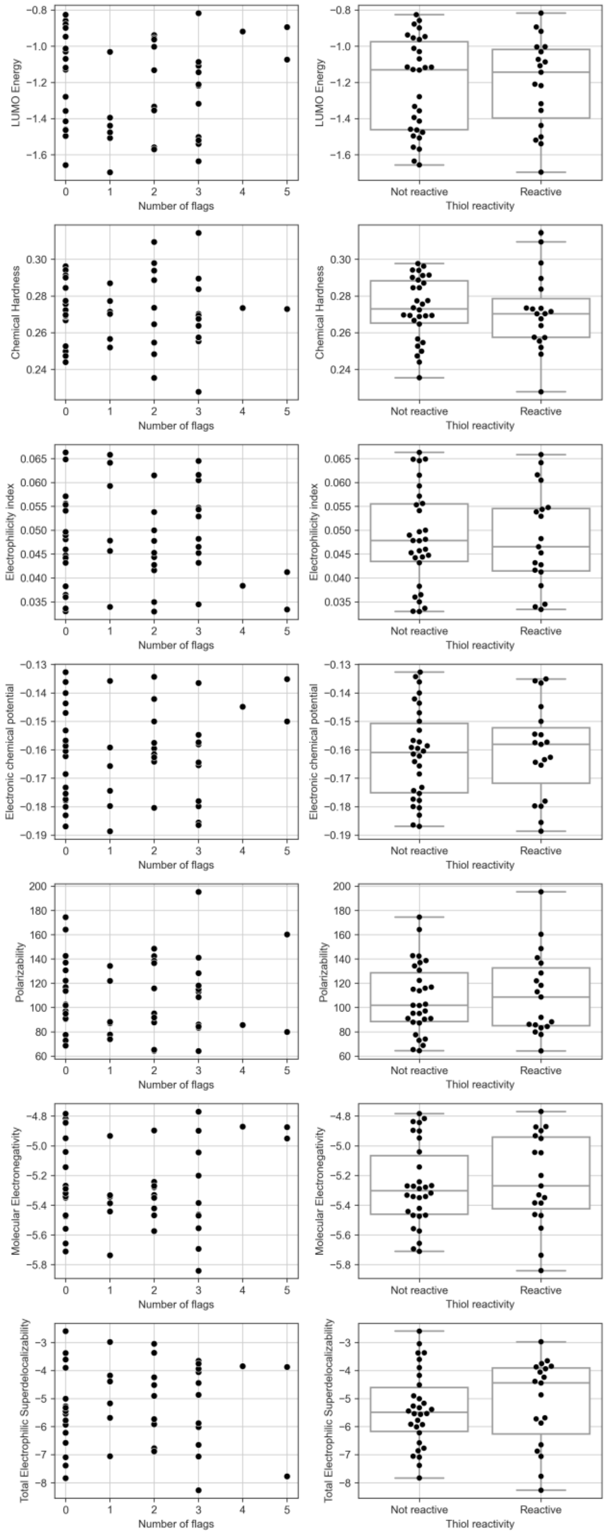

Figure S4. Relationship between selected descriptors derived from quantum-chemical calculation and reactivity parameters, i.e., the number of flags (left column) and thiol reactivity (right column).

2 Experimental procedures

No unexpected or unusually high safety hazards were encountered.

2.1 ChEMBL substructure search

The bioactive compounds were downloaded in sdf format from the ChEMBL database, which contained 2,331,700 compounds. The database was accessed on 2022-10-21, and a substructure search was performed using the KNIME analysis platform. First, structures were converted to RDKit format using the RDKit From Molecule node. Second, a substructure search was performed using the RDKit Substructure Filter. The SMARTS patterns used for the substructure search are listed in **Table S1**.

Table S1. SMARTS patterns used for the substructure search.

Substituent position	2	4	5
Any substituent		c1cncs1	
Amide	N(C=O)c1nccs1	c1nc(N(C=O))cs1	c1ncc(N(C=O))s1
Amine	[N;!H0;$(N-[#6]);!$(N-[!#6;!#1]);!$(N-C=[O,N,S])]c1nccs1	c1nc([N;!H0;$(N-[#6]);!$(N-[!#6;!#1]);!$(N-C=[O,N,S])])cs1	c1ncc([N;!H0;$(N-[#6]);!$(N-[!#6;!#1]);!$(N-C=[O,N,S])])s1
Primary amine	[N;H2;D1;$(N-!@[#6]);!$(N-C=[O,N,S])]c1nccs1	c1nc([N;H2;D1;$(N-!@[#6]);!$(N-C=[O,N,S])])cs1	c1ncc([N;H2;D1;$(N-!@[#6]);!$(N-C=[O,N,S])])s1
-COOR	[CX3](=O)([OX2H0][#6])c1nccs1	c1nc([CX3](=O)([OX2H0][#6]))cs1	c1ncc([CX3](=O)([OX2H0][#6]))s1
-COOH	C(=O)([O;H,-])c1nccs1	c1nc(C(=O)([O;H,-]))cs1	c1ncc(C(=O)([O;H,-]))s1
-Br	Brc1nccs1	c1nc(Br)cs1	c1ncc(Br)s1
-CN	N#Cc1nccs1	c1nc(C#N)cs1	c1ncc(C#N)s1

2.2 Enzymatic inhibition assays

2.2.1 MurA assay

A recombinant *E. coli* MurA enzyme (UDP-*N*-acetylglucosamine enolpyruvyl transferase) was expressed in *E. coli.*[1] In a colorimetric assay using malachite green, inhibition of the enzyme was monitored by following the orthophosphate formed during the enzymatic reaction. Briefly, compounds were pre-incubated at a concentration of 500 µM with MurA and the substrate UDP-*N*-acetylglucosamine with or without 1 mM DTT. The enzymatic reaction was initiated with the second substrate phosphoenolpyruvate and incubated for 15 min at 37 °C. Alternatively, the final

mixture was prepared without pre-incubation and incubated for 15 min at 37 °C. In both experiments, with and without 30 min of pre-incubation, the final mixture (50 μL) contained: 50 mM HEPES, pH 8.0, 0.005% [v/v] Triton X-114, 200 μM UDP-*N*-acetylglucosamine, 100 μM phosphoenolpyruvate, purified MurA (diluted in 50 mM HEPES, pH 7.8), and 500 μM of each tested compound dissolved in DMSO. The final DMSO concentration was 5% [v/v]. The reaction was terminated by the addition of Biomol® reagent (100 μL), and after 5 min absorbance was measured at 650 nm using Synergy H4 microplate reader (BioTek Instruments, Inc., USA). All experiments were performed in duplicate. RAs were calculated with respect to blank experiments without tested compounds and with 5% DMSO [v/v]. IC_{50} values and Hill coefficients were determined by measuring residual activities at 7 different compound concentrations and calculated using GraphPad Prism (GraphPad Software, San Diego, CA, USA).

2.2.2 DdlB assay

A recombinant *E. coli* DdlB enzyme (D-alanine:D-alanine ligase) was expressed in *E. coli.*[1] In a colorimetric assay using malachite green, inhibition of the enzyme was monitored by following the orthophosphate formed during the enzymatic reaction. Briefly, the final mixture (50 μL) contained: 50 mM HEPES, pH 8.0, 0.005% [v/v] Triton X-114, 5 mM $MgCl_2$, 6.5 mM $(NH_4)_2SO_4$, 10 mM KCl, 700 μM D-Ala, 100 μM ATP, purified DdlB (diluted in 50 mM HEPES, pH 8.0), and 500 μM of each tested compound dissolved in DMSO. The final DMSO concentration was 5% [v/v]. After 20 min of incubation at 37 °C, the reaction was terminated by the addition of Biomol® reagent (100 μL), and after 5 min at room temperature, absorbance was measured at 650 nm using Synergy H4 microplate reader (BioTek Instruments, Inc., USA). All experiments were performed in duplicate. RAs were calculated with respect to blank experiments without tested compounds and with 5% DMSO [v/v].

2.2.3 $3CL^{pro}$ assay

Codon optimized gene encoding SARS–CoV-2 $3CL^{pro}$ gene (Integrated DNA Technologies, USA) was cloned into pET-28c(+) plasmid and used to transform *E. coli* NiCo21(DE3) (New England Biolabs, USA). Transformed bacteria were grown at 37 °C and 250 rpm in LB broth supplemented with kanamycin until OD_{600} ~ 1.8. Cultures were chilled on ice for 10 min and expression was

induced with 0.2 mM IPTG. Expression was continued at 16 °C, 250 rpm for 24 h. Cells were harvested by centrifugation (2×10 min, 3000×*g*, 4 °C) and stored at –80 °C until purification of the recombinant $3CL^{pro}$.

Cell pellets were resuspended in buffer A (20 mM Tris, pH 7.5, 0.05 mM EDTA, 2.5 mM DTT, 10% glycerol) and lysed on ice by sonication. Cell debris was removed by centrifugation (2x30 min, 16000×*g*, 4 °C). Clear lysate was filtered through a 100-kDa MWCO unit (Amicon Ultra-15 centrifugal filter units; Merck, Germany). $(NH_4)_2SO_4$ (0.5 M final concentration) was slowly added to the filtrate which was then loaded onto a 1 mL HiTrap Phenyl HP column (Cytiva, USA) pre-equilibrated with buffer B (50 mM Tris, pH 7.5, 0.5 M $(NH_4)_2SO_4$, 0.05 mM EDTA, 2.5 mM DTT, 10% glycerol). The column was washed with 20 volumes of buffer B and the $3CL^{pro}$ was eluted with a linear gradient into buffer A. The eluted protein was concentrated with a 30-kDa MWCO unit (Ultra-4 centrifugal filter units; Amicon), snap-frozen in liquid nitrogen, and stored at −80 °C. Protein concentration was determined by absorbance at 280 nm and considering its extinction coefficient of 34380 M^{-1} cm^{-1}. Purity was assessed by SDS-PAGE.

The enzymatic activity of $3CL^{pro}$ was measured by kinetic assay using FRET fluorogenic substrate DABCYL-KTSAVLQSGFRKME-EDANS (CPC Scientific, CA, USA). Experiments were performed in assay buffer 50 mM Tris-HCl pH 7.3, 1 mM EDTA, 0.05% Triton X-114, with or without 1 mM DTT. Briefly, compounds were pre-incubated at a concentration of 500 µM with $3CL^{pro}$ for 30 min at 30 °C. The reaction was started by adding substrate, and the increase in fluorescence intensity was measured using Synergy H4 microplate reader (BioTek Instruments, Inc., USA) at λex = 360/40 nm and λem = 440/40 nm. The final concentrations were as follows: compound, 500 µM; substrate, 20 µM; $3CL^{pro}$, 50 nM; DMSO, 10% [v/v]. DMSO replaced the compound for the control experiments. For determination of *b* (blank), the enzyme was replaced with Tris-HCl buffered solution. Initial velocities (*v*) were calculated from the linear trends obtained, with each measurement performed in duplicate. Inhibitory potencies were expressed as residual activities, RA = $(v_i - b)/(v_o - b)$, where v_i is the velocity in the presence of the test compound, and v_0 is the control velocity in the presence of DMSO. To check for spectral interference, absorbance at the excitation and emission wavelengths, and autofluorescence were determined for the active compounds in buffer solution.

2.2.4 MetAP1a assay

The enzymatic activity of *M. tuberculosis* MetAP1a, was measured by a kinetic assay using a fluorogenic substrate L-methionine 7-amido-4-methylcoumarin as described previously.[2] Briefly, the final mixture (80 µL) contained: 40 mM HEPES pH 7.5, 100 mM NaCl, 100 µM L-methionine 7-amido-4-methylcoumarin, 200 nM $CoCl_2$, 200 nM MetAP1a, and 625 µM of the tested compound. The final concentration of DMSO was 1.25% [v/v]. The mixture was gently shaken and the increase in fluorescence intensity was measured using Synergy H4 microplate reader (BioTek Instruments, Inc., USA) at λex = 360 nm and λem = 460 nm. All experiments were performed in duplicate. RAs were calculated as the slope of the initial linear trend obtained for the measured compound divided by the initial linear trend of the blank experiment in which the compound was replaced with pure DMSO. To check for spectral interference, absorbance at the excitation and emission wavelengths, and autofluorescence were determined for active compounds in buffer solution.

2.3 Aqueous stability assay

The aqueous stability of all compounds was determined spectrophotometrically by following the changes in the absorption spectra of the compounds, as described previously.[3] Briefly, the final mixture contained: 50 mM Tris-HCl pH 7.4, 0.5 mM EDTA, and 50 µM of the tested compound. The final concentration of DMSO was 5% [v/v]. The mixtures were incubated in 96-well flat-bottom UV-transparent microplates (CLS3635, Corning, USA) without lids at 37 °C using Synergy H4 microplate reader (BioTek Instruments, Inc., USA) and the absorbance spectrum (244–400 nm) was acquired in sweep mode after 0, 15, 30, 60, 120, 180, and 240 min using a discontinuous kinetic procedure in Gen5 software (BioTek Instruments, Inc., USA). The time required to read the entire plate was 3 min. A blank experiment was performed without compound and the obtained baseline was subtracted from each measurement. Compounds with an absorbance maximum of less than 0.2 AU were assigned a low absorbance flag and were not evaluated due to the high experimental error in this assay. For other compounds, the relative absorbance difference between the first time point and 60 min at the most responsive wavelength was calculated. If the relative absorbance difference for the compound in the buffer was below 0.1, between 0.1–0.2, and above 0.2, the compound was classified as stable, intermediate, and unstable, respectively.

2.4 Redox activity assays

Assays were performed according to previously optimized procedures.[4] Experiments were performed in 96-well microplates in assay buffer (50 mM HEPES, 50 mM NaCl, pH 7.5). Threshold values for activity flags were above 10-fold standard deviation compared to DMSO blanks. All reagent solutions were freshly prepared before performing the experiments. 3-Methyltoxoflavin was used as a control compound.

2.4.1 HRP-PR assay

Briefly, to 58 µL of assay buffer, 10 µL of 2 mM compound DMSO stock solution, and 66 µL of assay buffer (redox-free) or 66 µL of 3 mM DTT in assay buffer were added. The solutions were incubated for 15 min at room temperature, then 66 µL of detection reagent (300 µg/mL phenol red and 180 µg/mL HRP [150–250 units/mg solid] in assay buffer) was added and the solutions were incubated for 5 min at room temperature. The final concentrations were: 100 µM compound, 100 µg/mL phenol red, 60 µg/mL HRP, 1 mM DTT, 5% [v/v] DMSO. The reaction was then quenched by the addition of 1 M NaOH (aq) (10 µL) and subsequently the absorbance was measured at 610 nm using Synergy H4 microplate reader (BioTek Instruments, Inc., USA). In a blank experiment, the compound solution was replaced with pure DMSO. The measured absorbance for each compound was then divided by the blank value.

2.4.2 H_2DCFDA assay

First, the probe H_2DCFDA was dissolved in DMSO and diluted to 500 µM with 0.01 M NaOH. The obtained solution was incubated for 30 min in the dark at room temperature to hydrolyze the ester. To 52.5 µL of assay buffer, 7.5 µL of 2 mM compound DMSO stock solution, 75 µL of assay buffer (redox-free) or 75 µL of 200 µM TCEP in assay buffer, and 15 µL of 500 µM H_2DCFDA were added. The final concentrations were: 100 µM compound, 50 µM H_2DCFDA, 100 µM TCEP, and 5% [v/v] DMSO. The microplate was covered with a lid and incubated for 30 min in the dark at room temperature. Fluorescence intensity was then measured using Synergy H4 microplate reader (BioTek Instruments, Inc., USA) at $\lambda ex = 485$ nm and $\lambda em = 535$ nm. In a blank experiment, the compound solution was replaced with pure DMSO. The measured fluorescence for each compound was then divided by the blank value. To check for spectral interference, absorbance at

the excitation and emission wavelengths, and autofluorescence were determined for active compounds in buffer solution.

2.4.3 *Resazurin assay*

Briefly, to 100 μL of assay buffer, 2 μL of 1 or 0.1 mM compound DMSO stock solution, and 100 μL of resazurin solution (10 μM resazurin and 200 μM DTT in assay buffer) were added. The final concentrations were: 1 or 10 μM compound, 5 μM resazurin, 100 μM DTT, and 1% [v/v] DMSO. The microplate was covered with a lid and incubated for 30 min in the dark at room temperature. Fluorescence intensity was then measured using Synergy H4 microplate reader (BioTek Instruments, Inc., USA) at λex = 560 nm and λem = 590 nm. In a blank experiment, the compound solution was replaced with pure DMSO. The measured fluorescence for each compound was then divided by the blank value. To check for spectral interference, absorbance at the excitation and emission wavelengths, and autofluorescence were determined for active compounds in buffer solution.

2.5 Thiol reactivity assays

Assays were performed according to previously optimized procedures.[4] Experiments were performed in duplicate in 96-well microplates in assay buffer (20 mM sodium phosphate, 150 mM NaCl, pH 7.4). All reagent solutions were freshly prepared before performing the experiments. 2-Chloro-*N*-(3-chlorophenyl)acetamide was used as a control compound.

2.5.1 *DTNB assay*

Briefly, 100 μM of compound was incubated at 37 °C in a mixture of 100 μM TCEP and 25 μM DTNB (which generates 50 μM TNB^{2-} *in situ*) in assay buffer containing 5 % final DMSO concentration. Absorbance at 412 nm was measured every 5 min for 4 h using Synergy H4 microplate reader (BioTek Instruments, Inc., USA) to monitor TNB^{2-} depletion. Compound background absorbance was subtracted from each measurement.

2.5.2 *TNB^{2-} assay*

Briefly, 100 or 200 μM of compound was incubated at 37 °C in a mixture of 50 or 100 μM TNB^{2-} in assay buffer containing 5 % final DMSO concentration. Absorbance at 412 nm was measured

every 5 min for 14–21 h using Synergy H4 microplate reader (BioTek Instruments, Inc., USA) to monitor TNB^{2-} depletion. When the reaction between the compound and TNB^{2-} was completed, 5 μL of TCEP was added to a final concentration of 200 μM and the reaction was monitored for up to 2 additional hours. To determine the baseline drift due to the oxidation of TNB^{2-} to DTNB, a blank experiment was performed by replacing the solution of the compound with pure DMSO. Compound background absorbance and baseline drift were subtracted from each measurement.

2.6 Quantum-mechanical calculations

The quantum-mechanical (QM) computations were done on a workstation computer (4 14-core Intel® Xeon® Gold 5120 2.20 GHz processors, 48 GB RAM, 2 TB SSD, running Ubuntu 20.04.3 LTS) with Schrödinger Small Molecule Discovery Suite Release 2021-1 (Schrödinger, LLC, New York, USA, 2021) using Jaguar[5] as the QM engine. The most probable protonation/tautomer states were determined by Epik at pH 7.0 ± 0.1,[6] and the structures minimized. A geometric optimization in water with subsequent properties calculation was first performed with semiempirical MOPAC2012 and NDDO[7] modules (PM7 with COSMO solvation – keywords: PM7, gnorm=0.0001, ddmin=0.0, scfcrt=1.D-9, eps=78.40, nspa=162, mullik, bonds, pi, super, plotden), then the optimized structures were used for further DFT calculations. A geometric optimization in water (Poisson-Boltzmann solver) at M06-2X-D3/LACVP**++ level of theory with ultrafine pseudospectral grids was followed by calculation of properties: Mulliken, Stockholder and ESP charges, Fukui indices, molecular orbitals (HOMO, LUMO), polarizability. Additionally, electron density, average local ionization energy (ALIE), noncovalent interactions, and electrostatic potential were calculated and plotted on a grid.

3 Chemistry

3.1 General chemistry

Compounds from the in-house chemical library and commercially available compounds were purchased from several vendors (AA Blocks, Acros, Apollo Scientific, ChemBridge, Enamine, Fluka, Fluorochem, Maybridge, Sigma-Aldrich, TCI, and Vitas-M) and used as received. The reagents and solvents were used as received from commercial suppliers. Reactions were monitored using analytical thin-layer chromatography on silica gel 60 F_{254} Al plates, and the components were visualized under UV light and/or through staining with the relevant reagent. Normal phase

flash column chromatography was performed with Merck Silica Gel 60 (particle size 0.040–0.063 mm; Merck, Germany). Melting points were determined on a Leica hot-stage microscope and are uncorrected. Nuclear magnetic resonance spectra were recorded on a Bruker Avance III 400 MHz spectrometer at 400 MHz for ^{1}H and 101 MHz for ^{13}C, using DMSO-d_6 or $CDCl_3$ with TMS as the internal standard, as solvents. Chemical shifts are reported in *parts per million* (ppm), TMS peak was calibrated to 0 ppm or, alternatively, the central peak of the residual solvent resonance was used as the internal standard. The multiplicities are reported as follows: *s* (singlet), *d* (doublet), *t* (triplet), *q* (quartet), *m* (multiplet), *dd* (doublet of doublets), *ddd* (doublet doublet of doublets), *td* (triplet of doublets), *qd* (quartet of doublets), and *br* (broad), number of equivalent nuclei (by integration), coupling constants (*J*) quoted in Hertz (Hz). ATR IR spectra were recorded on a FT-IR spectrometer (Thermo Nicolet Nexus 470 ESP). High-resolution mass measurements were performed on a Thermo Scientific Q Exactive Plus Hybrid Quadrupole Orbitrap mass spectrometer (Thermo Fisher Scientific, Waltham, MA, USA). IR spectra were acquired with Thermo Nicolet FT-IR spectrophotometer. HPLC analyses were performed on Thermo Scientific Dionex UltiMate 3000 modular system (Thermo Fisher Scientific Inc.) with Waters Acquity UPLC® HSS C18 SB column (2.1 × 50 mm, 1.8 µm) thermostated at 40 °C, injection volume, 1–5 µL; flow rate, 0.4 mL/min; detector λ, 220 and 254 nm or Agilent Eclipse XDB-C8 (4.6 × 150 mm, 3.5 µm) thermostated at 40 °C, injection volume, 5 µL; flow rate, 1.0 mL/min; detector λ, 220 and 254 nm; mobile phase A: 0.1% TFA (v/v) in water; mobile phase B: MeCN, C: 20 mM phosphate buffer (pH = 7.22). Method I: 0–7 min, 90–10% A, 10%–90% B; 7–8 min, 10% A, 90% B. Method II: 0–15 min, 90–0% A, 10%–100% B. Method III: 0–10 min, 10%–40% B, 90–60% C; 10–15 min, 40% B, 60% C.

3.2 General synthetic procedures

3.2.1 General procedure for bromination (general procedure 1)

Thiazole-2-amine (1.0 equiv.) was dissolved in MeCN (30 mL) on an ice bath. To a stirred solution, *tert*-butyl nitrite (1.2 equiv.) and $CuBr_2$ (1.2 equiv.) were added, and the mixture was stirred on an ice bath overnight. The reaction mixture was extracted with ethyl acetate (2 × 50 mL) and water (2 × 50 mL). The combined organic phases were washed with brine, dried over

anhydrous sodium sulfate, filtered, and volatile components evaporated in *vacuo* to afford the crude product which was purified with flash column chromatography.

3.2.2 General procedure for esterification (general procedure 2)

2-Bromothiazole (1.0 equiv.) was suspended in anhydrous MeOH (20 mL) and cooled on an ice bath. $SOCl_2$ (2.0 equiv.) was added dropwise and the mixture was refluxed overnight. The solvent was evaporated, the residue dissolved in water (2 mL) and neutralized with K_2CO_3. The aqueous phase was extracted with ethyl acetate (2 × 25 mL). The combined organic phases were washed with brine (50 mL), dried over anhydrous sodium sulfate, filtered, and volatile components evaporated in vacuo to afford the crude product that was purified with flash column chromatography.

3.2.3 General procedure for demethylation (general procedure 3)

Methyl 2-oxoacetate (1.0 equiv.) was suspended in THF (20 mL) and cooled on an ice bath. LiOH (0.1 M, 5.0 equiv.) was added, and the mixture was stirred on an ice bath for 2 hours. The reaction mixture was neutralized with NaOH (1 M, 1–5 mL) and the impurities were extracted with ethyl acetate (2 × 20 mL). The aqueous phase was acidified to pH 1 with HCl (1 M) and the product precipitated from the solution.

3.2.4 General procedure for acylation (general procedure 4)

2-Aminothiazole (1.0 equiv.) was dissolved in DCM (20 mL) under argon. Next, Et_3N (2.2–4.4 equiv.) was added and the reaction mixture was cooled on an ice bath. Methyl oxalyl chloride or methyl malonyl chloride (1.1–2.2 equiv.) was added dropwise and the reaction mixture was stirred under argon at room temperature overnight and extracted with DCM (50 mL), which was washed with saturated brine (2 × 15 mL). The combined organic phases were dried over anhydrous sodium sulfate, filtered, and volatile components evaporated in vacuo to afford the crude product.

3.3 Synthesis and characterization of compounds

3.3.1 2-Bromo-4-(3,4-difluorophenyl)thiazole (**19**)

Synthesized from 4-(3,4-difluorophenyl)thiazol-2-amine (1.0 g, 3.26 mmol, 1.0 equiv.) via general procedure 1, with some modifications: *tert*-butyl nitrite (0.7 equiv.) and $CuBr_2$ (0.2 equiv.). The

purification was carried out using (EtOAc/hexane: 1/1, v/v) to afford **19** as a white solid in 0.1% yield (11 mg). R_f = 0.62 (EtOAc/hexane: 1:1, v/v). mp = 52–53 °C. ^{1}H NMR (400 MHz, $CDCl_3$): δ (ppm) = 7.18 (ddd, *J* = 10.1, 8.6, 8.1 Hz, 1H), 7.35 (s, 1H), 7.55 (dddd, *J* = 8.7, 4.3, 2.2, 1.4 Hz, 1H), 7.67 (ddd, *J* = 11.4, 7.6, 2.2 Hz, 1H). ^{13}C NMR (100 MHz, $CDCl_3$): δ (ppm) = 115.5 (d, $J_{C,F}$ = 18.9 Hz), 116.7 (d, $J_{C,F}$ = 1.5 Hz), 117.8 (d, $J_{C,F}$ = 17.8 Hz), 122.3 (dd, $J_{C,F}$ = 6.4, 3.6 Hz), 130.4 (dd, $J_{C,F}$ = 6.4, 3.9 Hz), 136.4 (s), 149.1–152.2 (m, 2C), 153.7 (d, $J_{C,F}$ = 2.1 Hz). HRMS (ESI+): *m/z* calcd for $C_9H_5BrF_2NS$ $[M+H]^+$ 275.9289; found 275.9286. HPLC purity, 91.4% (method I, t_R = 5.33 min).

*3.3.2 Methyl 2-bromothiazole-5-carboxylate (**20**)*

Synthesized from 2-bromothiazole-5-carboxylic acid (0.5 g, 2.4 mmol, 1.0 equiv.) via general procedure 2, where H_2SO_4 (256 µL, 4.81 mmol, 2 equiv.) was used instead of $SOCl_2$. The purification was carried out using (EtOAc/hexane: 1/4, v/v) to afford **20** as orange crystals in 70% of yield. R_f = 0.33 (EtOAc/hexane: 1/4, v/v). mp = 52–53 °C. ^{1}H NMR (400 MHz, $CDCl_3$): δ (ppm) = 3.91 (s, 3H), 8.16 (s, 1H). ^{13}C NMR (100 MHz, $CDCl_3$): δ (ppm) = 52.9, 132.8, 142.2, 148.1, 160.5. HRMS (ESI+): *m/z* calcd for $C_5H_5BrNO_2S$ $[M+H]^+$ 221.9219; found 221.9217. HPLC purity, 95.3% (method I, t_R = 3.32 min).

*3.3.3 Methyl 2-bromothiazole-4-carboxylate (**21**)*

Synthesized from 2-bromothiazole-4-carboxylic acid (1.0 g, 4.8 mmol, 1.0 equiv.) via general procedure 2. The purification was carried out using (EtOAc/hexane: 1/1, v/v) to afford **21** as white crystals in 50% of yield. R_f = 0.0.46 (EtOAc/hexane: 1/1, v/v). mp = 97–98 °C. ^{1}H NMR (400 MHz, $CDCl_3$): δ (ppm) = 3.93 (s, 3H), 8.08 (s, 1H). ^{13}C NMR (100 MHz, $CDCl_3$): δ (ppm) = 52.8, 129.4, 145.4, 152.8, 160.8. HRMS (ESI–): *m/z* calcd for $C_5H_3BrNO_2S$ $[M–H]^–$ 219.9073; found 219.9070. HPLC purity, 98.9% (method I, t_R = 2.31 min).

*3.3.4 Ethyl 2-bromothiazole-5-carboxylate (**22**)*

Synthesized from ethyl 2-aminothiazole-5-carboxylate (3 g, 17.4 mmol, 1.0 equiv.) via general procedure 1. The purification was carried out using (EtOAc/hexane: 1/6, v/v) to afford **22** as a colorless oil in 4.4% of yield (18.2 mg). R_f = 0.35 (EtOAc/hexane: 1/6, v/v). ^{1}H NMR (400 MHz,

$CDCl_3$): δ (ppm) = 1.38 (t, J = 7.1 Hz, 3H), 4.37 (q, J = 7.1 Hz, 2H), 8.15 (s, 1H). ^{13}C NMR (100 MHz, $CDCl_3$): δ (ppm) = 14.27, 62.08, 133.27, 141.98, 147.80, 159.99. HRMS (ESI+): *m/z* calcd for $C_6H_7BrNO_2S$ $[M+H]^+$ 235.9375; found 235.9378. HPLC purity, 98.2% (method I, t_R = 4.01 min).

*3.3.5 Ethyl 2-bromothiazole-4-carboxylate (**23**)*

Synthesized from ethyl 2-aminothiazole-4-carboxylate (2.25 g, 13.1 mmol, 1.0 equiv.) via general procedure 1. The purification was carried out using (EtOAc/hexane: 1/4, v/v) to afford **23** as a white solid in 53% of yield (1.53 g). R_f = 0.33 (EtOAc/hexane: 1/4, v/v). mp = 52–53 °C. ^1H NMR (400 MHz, $CDCl_3$): δ (ppm) = 1.36 (t, J = 7.1 Hz, 3H), 4.38 (q, J = 7.1 Hz, 2H), 8.09 (s, 1H). ^{13}C NMR (100 MHz, $CDCl_3$): δ (ppm) = 14.36, 61.91, 130.92, 136.85, 147.26, 160.15. HRMS (ESI+): *m/z* calcd for $C_6H_7BrNO_2S$ $[M+H]^+$ 235.9375; found 235,9372. HPLC purity, 96.9% (method I, t_R = 3.15 min).

*3.3.6 2-((5-Bromothiazol-2-yl)amino)-2-oxoacetic acid (**26**)*

Synthesized from methyl 2-((5-bromothiazol-2-yl)amino)-2-oxoacetate (100 mg, 0.38 mmol, 1.0 equiv.) via general procedure 3. Compound **26** was obtained as light brown solid in 23% of yield (24 mg). R_f = 0 (DCM/MeOH, 20:1, v/v). ^1H NMR (400 MHz, DMSO-d_6): δ (ppm) = 13.16 (s, 1H), 7.68 (s, 1H). ^{13}C NMR (100 MHz, DMSO-d_6): δ (ppm) = 165.54, 160.28, 157.52, 139.31, 103.01. HRMS (ESI–): *m/z* calcd for $C_5H_2O_3N_2BrS$ $[M-H]^-$ 248.8975; found 248.8972. HPLC purity, 93.4% (method II, t_R = 2.10 min).

*3.3.7 Methyl 3-((5-bromothiazol-2-yl)amino)-3-oxopropanoate (**27**)*

Synthesized from 5-bromothiazol-2-amine (1.0 g, 3.85 mmol, 1.0 equiv.), Et_3N (2.4 mL, 16.9 mmol, 4.4 equiv.) and methyl malonyl chloride (0.9 mL, 8.5 mmol, 2.2 equiv.) via general procedure 4. The purification was carried out using recrystallization from EtOAc (10 mL) with a few drops of hexane and subsequent flash column chromatography (DCM/MeOH: 50/1, v/v) to afford **27** as a white solid in 32% yield. R_f = 0.34 (DCM/MeOH: 50/1, v/v). mp = 114–118 °C. ^1H NMR (400 MHz, DMSO-d_6): δ (ppm) = 12,59 (s, 1H), 7.58 (s, 1H), 3.65 (s, 3H), 3.62 (s, 2H). ^{13}C NMR (100 MHz, DMSO-d_6): δ (ppm) = 167.3, 164.7, 158.9, 138.9, 102.0, 52.2, 41.7. HRMS

(ESI+): *m/z* calcd for $C_7H_8O_3N_2BrS$ $[M+H]^+$ 278.9433; found 278.9429. HPLC purity, 94.8% (method II, t_R = 9.42 min).

3.3.8 *Methyl 2-((5-bromothiazol-2-yl)amino)-2-oxoacetate (**28**)*

5-Bromothiazol-2-amine bromide (1 g, 3.85 mmol, 1.0 equiv.) was dissolved in DCM (40 mL) under argon. Next, Et_3N (1.18 mL, 8.46 mmol, 2.2 equiv.) was added and the reaction mixture was cooled on an ice bath. Methyl oxalyl chloride (0,4 mL, 4,2 mmol; 1,1 equiv.) was added dropwise and the reaction mixture was stirred under argon at room temperature overnight and extracted with DCM (100 mL) which was washed with brine (2 × 15 mL). The combined organic phases were dried over anhydrous sodium sulfate, filtered, and volatile components evaporated in vacuo to afford **28** as pale-yellow solid in 50% of yield (0.54 g). R_f = 0.63 (DCM/MeOH: 20/1, v/v). ^{1}H NMR (400 MHz, DMSO-d_6): δ (ppm) = 13.29 (s, 1H), 7.65 (s, 1H), 3.82 (s, 3H). ^{13}C NMR (100 MHz, DMSO-d_6): δ (ppm) =159.2, 157.6, 156.2, 139.2, 103.4, 53.4. HRMS (ESI+): *m/z* calcd for $C_6H_6O_3N_2BrS$ $[M+H]^+$ 264.9277; found 264.9277. HPLC purity, 94.8% (method II, t_R = 9.42 min).

3.3.9 *2-(2-Bromothiazol-4-yl)acetic acid (**29**)*

Methyl 2-(2-bromothiazol-4-yl)acetate (0.54 g, 2.3 mmol, 1.0 equiv.) was dissolved in MeOH (2 mL), on an ice bath. NaOH (2 M, 2.0 equiv.) was added to the solution and stirred for 2 h at room temperature. The mixture was neutralized with HCl (1 M, 1 mL) and MeOH was evaporated. The reaction mixture was extracted with ethyl acetate (3 × 50 mL). The combined organic phases were washed with brine (100 mL), dried over anhydrous sodium sulfate, filtered, and volatile components evaporated in vacuo to afford the crude product which was purified with flash column chromatography to afford **29** as a white crystalline solid in 76% of yield (388 mg). mp = 120–122 °C. ^{1}H NMR (400 MHz, $CDCl_3$): δ (ppm) = 3.87 (d, *J* = 0.9 Hz, 2H), 7.19 (t, *J* = 0.9 Hz, 1H). ^{13}C NMR (100 MHz, $CDCl_3$): δ (ppm) = 36.5, 120.7, 136.2, 148.6, 174.8. HRMS (ESI+): *m/z* calcd for $C_5H_5BrNO_2S$ $[M+H]^+$ 221.9219; found 221.9215. HPLC purity, 97.7% (method I, t_R = 1.74 min).

3.3.10 *Methyl 3-((5-cyanothiazol-2-yl)amino)-3-oxopropanoate (**40**)*

Synthesized from 2-aminothiazole-5-carbonitrile (0.5 g, 4.0 mmol, 1.0 equiv.) and methyl malonyl chloride (0.47 mL, 4.4 mmol, 1.1 equiv.) via general procedure 4. The purification was carried out

using flash column chromatography (EtOAc/hexane: 1/1, v/v) to afford **40** as a pale-yellow solid in 15% yield (136 mg). R_f = 0.28 (EtOAc/hexane: 1/1, v/v). mp = 136–140 °C. IR (ATR): 3099, 2941, 2259, 2221, 2168, 1733, 1694, 1536, 1501, 1438, 1340, 1255, 1193, 1150, 1016, 898, 793, 709, 638, 547 cm^{-1}. ^1H NMR (400 MHz, DMSO-d_6): δ (ppm) = 13.14 (s, 1H), 8.39 (s, 1H), 3.69 (s, 2H), 3.66 (s, 3H). ^{13}C NMR (100 MHz, DMSO-d_6): δ (ppm) = 167.1, 165.1, 161.7, 150.2, 113.3, 98.0, 52.3, 41.8. HRMS (ESI+): *m/z* calcd for $C_8H_8O_3N_3S$ $[M+H]^+$ 226.0281; found 226.0284. HPLC purity, 98.4% (method II, t_R = 7.42 min).

*3.3.11 3-((5-Cyanothiazol-2-yl)amino)-3-oxopropanoic acid (**41**)*

Synthesized from methyl 3-((5-cyanothiazol-2-yl)amino)-3-oxopropanoate (100 mg, 0.44 mmol, 1.0 equiv.) via general procedure 3. Compound **41** was obtained as a white solid in 70% of yield (76 mg). R_f = 0 (DCM/MeOH: 20/1, v/v). IR (ATR): 3181, 3088, 2971, 2479, 2220, 1720, 1693, 1566, 1513, 1460, 1408, 1337, 1195, 1142, 988, 947, 883, 819, 740, 691, 650, 592, 545 cm^{-1}. ^1H NMR (400 MHz, DMSO-d_6): δ (ppm) = 13.04 (s, 1H), 12.91 (s, 1H), 8.36 (s, 1H), 3.54 (s, 2H). ^{13}C NMR (100 MHz, DMSO-d_6): δ (ppm) = 168.2, 166.1, 161.8, 150.2, 113.3, 97.8, 42.4. HRMS (ESI+): *m/z* calcd for $C_7H_6O_3N_3S$ $[M+H]^+$ 212.0124; found 212.0122. HPLC purity, 95.9% (method III, t_R = 8.10 min).

*3.3.12 2-((5-Cyanothiazol-2-yl)amino)-2-oxoacetic acid (**42**)*

Methyl 2-((5-cyanothiazol-2-yl)amino)-2-oxoacetate was synthesized from 2-aminothiazole-5-carbonitrile (0.5 g, 4.0 mmol, 1.0 equiv.), Et_3N (1.23 mL, 8.8 mmol, 2.2 equiv.) and methyl oxalyl chloride (0.47 mL, 4.4 mmol, 1.1 equiv.) via general procedure 4 to afford methyl 2-((5-cyanothiazol-2-yl)amino)-2-oxoacetate as a pale-yellow solid in 26% yield (227 mg). R_f = 0.58 (DCM/MeOH: 20/1, v/v). mp = 126–128 °C. IR (ATR): 3122, 2957, 2259, 2223, 1739, 1696, 1533, 1499, 1441, 1295, 1194, 1152, 1042, 972, 901, 790, 737, 631, 548 cm^{-1}. ^1H NMR (400 MHz, DMSO-d_6): δ (ppm) = 13.81 (s, 1H), 8.48 (s, 1H), 3.89 (s, 3H). ^{13}C NMR (100 MHz, DMSO-d_6): δ (ppm) = 161.8, 158.8, 156.9, 150.3, 113.1, 98.9, 53.5. HRMS (ESI+): *m/z* calcd for $C_7H_6O_3N_3S$ $[M+H]^+$ 212.0124; found 212.0119. HPLC purity, 91.1% (method II, t_R = 9.64 min).

Next, **42** was synthesized from methyl 2-((5-cyanothiazol-2-yl)amino)-2-oxoacetate (100 mg, 0.47 mmol, 1.0 equiv.) via general procedure 3. **42** was obtained as a white solid in 53% of yield (57

mg). R_f = 0 (DCM/MeOH: 20/1, v/v). IR (ATR): 3122, 2928, 2530, 2249, 2198, 1738, 1696, 1531, 1495, 1443, 1304, 1244, 1191, 1147, 886, 793, 715, 634, 549 cm^{-1}. ^{1}H NMR (400 MHz, DMSO-*d*$_6$): δ (ppm) = 13.57 (s, 1H), 8.48 (s, 1H). ^{13}C NMR (100 MHz, DMSO-*d*$_6$): δ (ppm) = 161.6, 159.9, 158.6, 150.4, 113.1, 98.9. HRMS (ESI–): *m/z* calcd for $C_6H_2O_3N_3S$ $[M–H]^-$ 195.9822; found 195.9815. HPLC purity, 97.9% (method III, t_R = 10.45 min).

4 References

(1) Proj, M.; Bozovičar, K.; Hrast, M.; Frlan, R.; Gobec, S. DNA-Encoded Library Screening on Two Validated Enzymes of the Peptidoglycan Biosynthetic Pathway. *Bioorg. Med. Chem. Lett.* **2022**, *73*, 128915. https://doi.org/10.1016/j.bmcl.2022.128915.

(2) Juhás, M.; Pallabothula, V. S. K.; Grabrijan, K.; Šimovičová, M.; Janďourek, O.; Konečná, K.; Bárta, P.; Paterová, P.; Gobec, S.; Sosič, I.; Zitko, J. Design, Synthesis and Biological Evaluation of Substituted 3-Amino-N-(Thiazol-2-Yl)Pyrazine-2-Carboxamides as Inhibitors of Mycobacterial Methionine Aminopeptidase 1. *Bioorganic Chem.* **2022**, *118*, 105489. https://doi.org/10.1016/j.bioorg.2021.105489.

(3) Kollár, L.; Gobec, M.; Proj, M.; Smrdel, L.; Knez, D.; Imre, T.; Gömöry, Á.; Petri, L.; Ábrányi-Balogh, P.; Csányi, D.; Ferenczy, G. G.; Gobec, S.; Sosič, I.; Keserű, G. M. Fragment-Sized and Bidentate (Immuno)Proteasome Inhibitors Derived from Cysteine and Threonine Targeting Warheads. *Cells* **2021**, *10* (12), 3431. https://doi.org/10.3390/cells10123431.

(4) Proj, M.; Knez, D.; Sosič, I.; Gobec, S. Redox Active or Thiol Reactive? Optimization of Rapid Screens to Identify Less Evident Nuisance Compounds. *Drug Discov. Today* **2022**, *27* (6), 1733–1742. https://doi.org/10.1016/j.drudis.2022.03.008.

(5) Bochevarov, A. D.; Harder, E.; Hughes, T. F.; Greenwood, J. R.; Braden, D. A.; Philipp, D. M.; Rinaldo, D.; Halls, M. D.; Zhang, J.; Friesner, R. A. Jaguar: A High-Performance Quantum Chemistry Software Program with Strengths in Life and Materials Sciences. *Int. J. Quantum Chem.* **2013**, *113* (18), 2110–2142. https://doi.org/10.1002/qua.24481.

(6) Greenwood, J. R.; Calkins, D.; Sullivan, A. P.; Shelley, J. C. Towards the Comprehensive, Rapid, and Accurate Prediction of the Favorable Tautomeric States of Drug-like Molecules in Aqueous Solution. *J. Comput. Aided Mol. Des.* **2010**, *24* (6), 591–604. https://doi.org/10.1007/s10822-010-9349-1.

(7) Stewart, J. J. MOPAC2012. Stewart Computational Chemistry, Colorado Springs. *CO USA* **2012**.
